# NMR of Natural Products as Potential Drugs

**DOI:** 10.3390/molecules26123763

**Published:** 2021-06-21

**Authors:** Poul Erik Hansen

**Affiliations:** Department of Science and Environment, Roskilde University, Universitetsvej 1, DK-4000 Roskilde, Denmark; Poulerik@ruc.dk

**Keywords:** natural products, hydrogen bonding, isotope effects on chemical shifts, tautomerism, DFT calculations, biological effects

## Abstract

This review outlines methods to investigate the structure of natural products with emphasis on intramolecular hydrogen bonding, tautomerism and ionic structures using NMR techniques. The focus is on 1H chemical shifts, isotope effects on chemical shifts and diffusion ordered spectroscopy. In addition, density functional theory calculations are performed to support NMR results. The review demonstrates how hydrogen bonding may lead to specific structures and how chemical equilibria, as well as tautomeric equilibria and ionic structures, can be detected. All these features are important for biological activity and a prerequisite for correct docking experiments and future use as drugs.

## 1. Introduction

For many years natural products have been a valuable source for new drugs. However, in recent years, this has diminished. An excellent overview dealing with this trend is given by Newman and Cragg [1]. This is a sad tendency. The use of natural products to cure illness has become even more important, as the price of developing new drugs has become enormous and, in this way, prohibitive for development of drugs to cure rare deceases. Natural products are used extensively in folk medicine. Many natural products are also used as building blocks in the synthesis of drugs. A famous example is taxol [2].

Several reviews exist, covering natural products and drugs in some way [3,4,5,6,7,8]. As many natural products have some sort of biological effect, not all can be included. Examples are presented to illustrate the theme of the paper. In addition, the present review will not deal with assignment of NMR spectra of natural products based on 2D NMR techniques, as this topic has been covered recently [9,10] and has been described in a very illustrative way [11,12]. Furthermore, techniques to determine the skeleton are not central to this review; the focus will be on other NMR techniques that can help describe important features, such as intramolecular hydrogen bonding, tautomerism, zwitter ion formation and functional groups, the functionality of which may vary depending on the physiological conditions. The use of NMR chemical shifts, isotope effect on chemical shifts, chemical shifts and coupling constants, mainly from an experimental view, but also in special cases based on theoretical calculations, will be treated.

The structure of the paper is as follows: at first, the just mentioned focus points are discussed and, at the end, a number of cases will be discussed, illustrating these points.

## 2. Intramolecular Hydrogen Bonding

**Figure 1:**
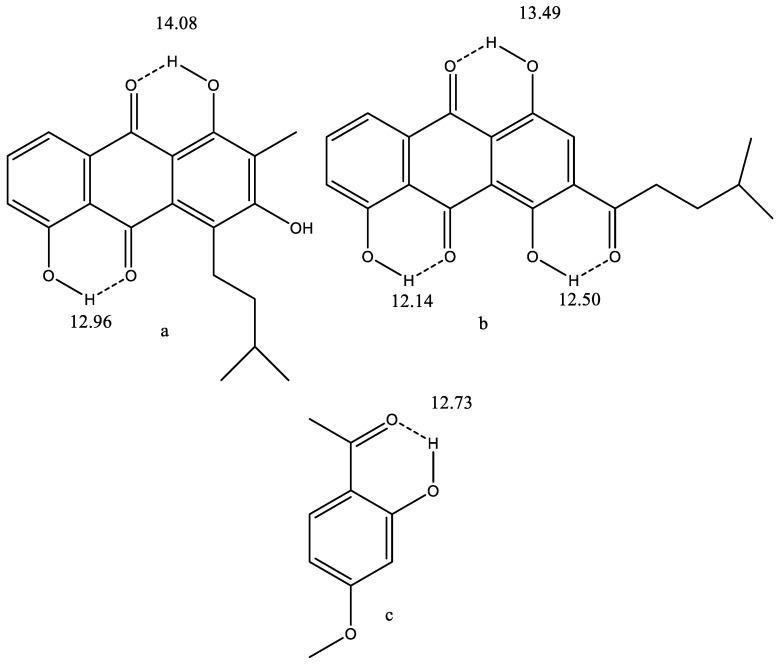
(**a**,**b**) Antitumor anthraquinones isolated from sea anemones. From Ref. [16]. (**c**) Paeonol from Ref. [17]. The numbers are OH chemical shifts.

A drug has to be able to pass the gut membrane. This ability is normally judged by log P defined as log ([solute]._oct_/[solute]._water_), oct being octanol and the solute assumed to be un-ionized. A positive value means it is more lipophilic and membrane-like. Calculational approaches have also been presented [13]. For compounds with polar substituents, intramolecular hydrogen bonds may be of importance, as the latter changes the polarity of the molecule and alters its possibility to pass membranes. An important factor is the strength of hydrogen bonds, as this determines the ability to maintain the hydrogen bond in hydrogen bonding solvents. The strength can be estimated using XH chemical shifts, X = O, S, N. The larger the chemical shift, the stronger the hydrogen bond (see Figure 1). Another possibility is the deuterium isotope effects on ^13^C and ^15^N chemical shifts [14]. The two parameters, XH chemical shifts and deuterium isotope effects on ^13^C chemical shifts, are related. The use of isotope effects has the advantage that isotope effects are obtained as a difference (see Section 3) and, in this way, substituent effects and solvent effects are eliminated [15]. Examples of intramolecular hydrogen bonding are seen in Figure 1a–c.

The large OH chemical shifts indicate rather strong hydrogen bonds and define the orientation of the side-chain in Figure 1b.

Paeonol (2-hydroxy-4-methoxyacetophenone) (Figure 1c) is found in peonies, such as Paeonia suffruticosa (moutan cortex), in Arisaema erubescens and in Dioscorea japonica. It is used in traditional Chinese medicine. Recent theoretical studies show that the intramolecular hydrogen bond is stable in water [17].

Molecules with intramolecular hydrogen bonds may also be tautomeric (see Section 4). This is more likely if OH groups, rather than NH groups, are involved.

## 3. Isotope Effects on Chemical Shifts

### 3.1. Secondary Isotope Effects

**Figure 2:**
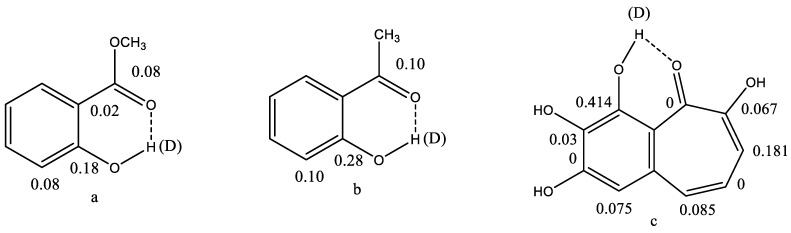
Deuterium isotope effects on ^13^C chemical shifts in ppm. Methyl salicylate (**a**) and *o*-hydroxyacetophenone (**b**). Taken from Ref. [14]. Purpurogallin (**c**). Only isotope effects due to the included deuterium are shown for clarity. In (**c**), the other OH groups are, of course, also deuterated. From Ref. [14].

Secondary isotope effects on chemical shifts stem from the difference in zero point energies of the Yl and the Yh species, Y being the nucleus under investigation and l and h being the light and heavy isotopes; H and D = ^2^H, as an example. The secondary isotope effects are defined as ^n^ΔY = δY(l) − δY(h), n being the number of bonds between the isotope and Y. The description so far covers intrinsic isotope effects. The intrinsic effect falls off very quickly in aliphatic systems, whereas they may be long range in conjugated systems. The stronger the hydrogen bond the larger the isotope effects; compare a and b in Figure 2. Figure 2a is methyl salicylate, also known as oil of wintergreen. The ester is clearly the poorest hydrogen bond acceptor.

In Figure 2b, a stronger hydrogen bond and a transmission of the isotope effect via the hydrogen bond is seen, as compared to Figure 2a. In Figure 2c the two-bond isotope effect is larger, as the heavy atom distance is smaller than in Figure 2b. Extensive transmission of isotope effects caused by conjugation can be seen. However, no isotope effect is seen at the carbonyl carbon. In case of a tautomeric equilibrium, such as in tropolone, one would expect a large equilibrium isotope effect due to the large chemical shift difference between the OH and the C=O carbon chemical shifts (see Equation (1), below).

**Figure 3:**
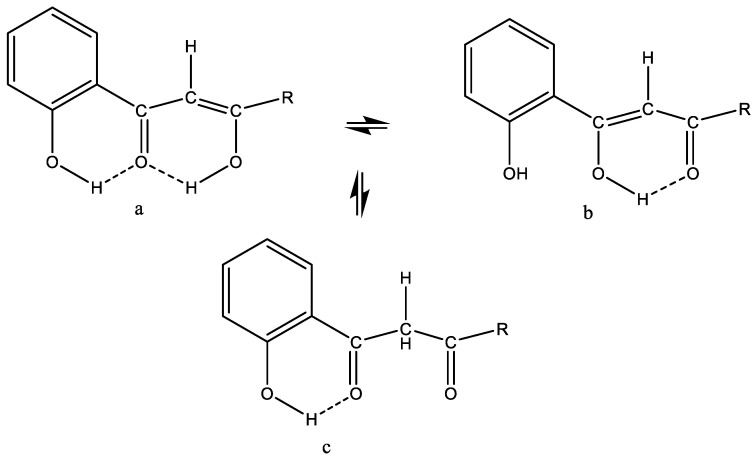
Tautomeric equilibrium of a β-diketone system. (**a**,**b**) are in fast equilibrium. (**c**) the keto form is in a slow equilibrium with the enol forms.

In addition to this, an equilibrium isotope effect may occur in tautomeric cases, so the full isotope effect for a tautomeric equilibrium is defined as:

ΔC=O(XD) = Δx * (δC=O − δCOH) + x * ^4^ΔCOH(XD)_int_ + (1 − x) * ^2^ΔC=O(XD)_int_ (1)

where x is the mole fraction, Δx is the change in the mole fraction upon deuteriation and δC=O − δCOH is the difference between the two carbons, the C=O and the C-OH carbons. For an example, see Figure 3a,b.

**Figure 4:**
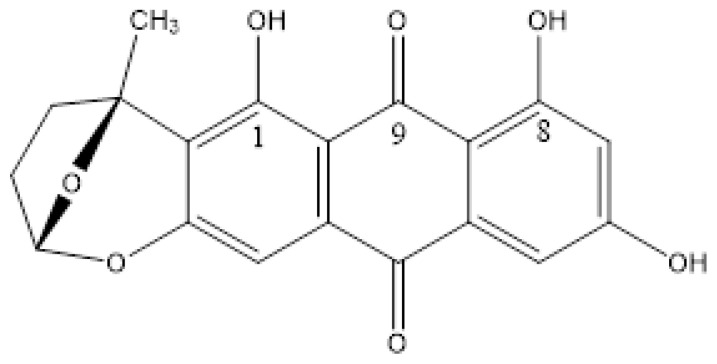
One-bond ^18^O isotope effects on ^13^C chemical shifts of averufin. The isotope effects are: C1 11ppb, C8 10 ppb and C9 29 ppb. Taken from Ref. [20]. Averufin is isolated for Aspergillus versicolor and it displays antibiotic properties.

In addition to deuterium isotope effects, heavy atom isotope effects can also be observed. ^18^O isotope effects on ^13^C chemical shifts have been measured in many natural products and have been used to elucidate biosynthetic pathways [18]. These effects are small, due to the small difference in mass between ^18^O and ^16^O. The trends for C=O bonds are clear; the higher the bond order, the larger is the isotope effect [19]. In addition, hydrogen bonding of intramolecular hydrogen bonds of RAHB type diminish the isotope effect, as the resonance form b leads to a lower C=O bond order. For an example, see Figure 4. See also discussion of tetracycline (Section 7.2.3).

### 3.2. Primary Isotope Effects

^P^Δ = δ_light_ − δ_heavy_ light is typically H and heavy is typically deuterium, but can also be tritium, ^3^H. Unfortunately, deuterium NMR resonances are often broad, so, although called primary, they are not so common. Tritium will give rise to larger effects, but is, of course, radioactive, so special precautions are needed. A general rule by Forsén et al. states that if the primary isotope effect is small and negative, a single hydrogen potential is present. If the isotope effect is small and positive, a weak hydrogen bond is at hand, whereas if it is large and positive a strong hydrogen bond is present [21]. Using these rules, one has to be sure that no equilibrium is taking place, as this will influence the magnitude of the primary isotope effects [22].

**Figure 5:**
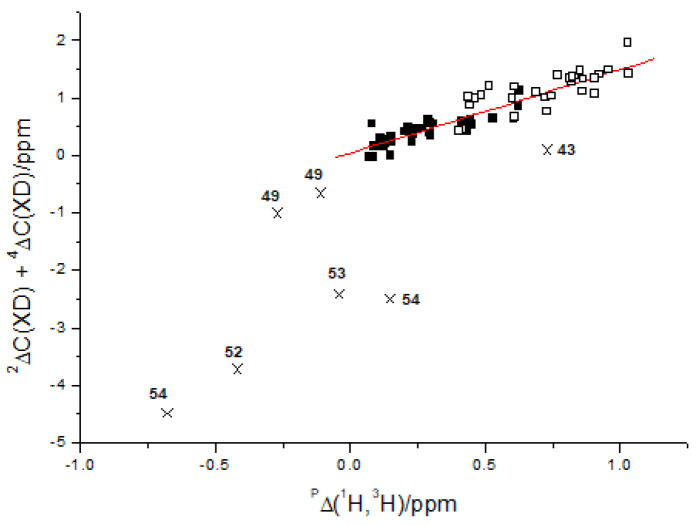
Plot of the sum of two- and four-bond deuterium isotope effects on ^13^C chemical shifts vs. primary tritium isotope effects. Taken from Ref. [22], with permission from John Wiley and Sons.

Figure 5 shows that the sum of two- and four-bond deuterium isotope effects on ^13^C chemical shifts vs. primary tritium isotope effects can be correlated for “static” hydrogen bonded systems, whereas the tautomeric cases marked with crosses fall off that correlation line. The primary isotope effects could also have been primary deuterium isotope effects.

## 4. Tautomerism

To be able to determine the correct form of a drug molecule is very important, as drugs often bind to receptors with a high degree of specificity. This is the case for tautomeric molecules, which may take up different forms, often depending on the solvent [23]. If the barrier to interconversion for a tautomeric molecule is low, the predominant form is of little importance. A classic example is the enol conversion of a β-diketone (see Figure 3), as seen between forms a and b. In contrast, the barrier between the enol forms a, b and the keto form c is normally high, leading to observation by NMR of two different forms. Examples to be discussed later are curcumin (Section 7.1), tetracycline (Section 7.2.3) usnic acid (Section 7.2.2) and garcinia derivatives (Section 7.2.4).

### 4.1. Isotope Effects

A useful method to detect tautomerism is to analyze deuterium isotope effects on ^13^C chemical shifts. In that case, the “tautomeric” proton is partly exchanged with deuterium. This leads to a mix of intrinsic and equilibrium isotope effects (see Equation (1)). As the equilibrium effect depends on the chemical shift difference of a given nucleus in the two tautomers (see Equation (1)), the equilibrium isotope effects may be observed far from the site of deuteriation. 

### 4.2. Coupling Constants

**Figure 6:**
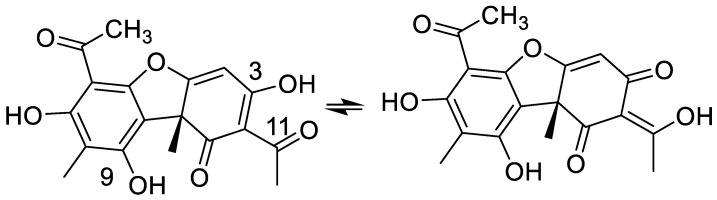
Tautomeric equilibrium of β-triketone type, usnic acid.

If a hydrogen substituted nitrogen is part of the tautomeric equilibrium, ^1^J(NH) is an excellent probe for tautomerism [24]. A situation more often occurring is that an OH group is part of the tautomeric equilibrium. In this case, the ^2^J(C,OH) coupling is a very good probe. An example is usnic acid (Figure 6). 

The two two-bond couplings for usnic acid are as follows: ^2^J(C-11,OH) = 3.5 Hz and ^2^J(C-3,OH) = 3.6 Hz, indicating an equilibrium close to 50:50, in agreement with the isotope effects results [25]. When the OH group at position 9 is substituted, the coupling constants change to ^2^J(C-11,OH) = 3.7 Hz and ^2^J(C-3,OH) = 3.2 Hz, illustrating a change in the equilibrium [25]. 

### 4.3. Chemical Shifts

In the study of tautomeric equilibria, it can be an advantage to study nuclei with a large chemical shift range, nuclei such as ^15^N or ^17^O. The use of the latter has been reviewed [26,27]. The hydrogen bond in curcumin (see Section 7.1) has been studied in the solid state by ^17^O NMR [28]. Other β-diketones have also been investigated in the liquid state [29,30]. ^15^N chemical shifts in relation to equilibrium have been reviewed [24]. A common problem in using chemical shifts to estimate the position of a tautomeric equilibrium is the lack of knowledge of the chemical shifts of both tautomers. In some cases, information for one of tautomers can be obtained from solid state NMR. In that case, density functional theory (DFT) calculations (see Section 6) can provide the chemical shifts of the unknown tautomer, or for both, if solid state NMR spectra are not available. 

## 5. Diffusion Experiments

**Figure 7:**
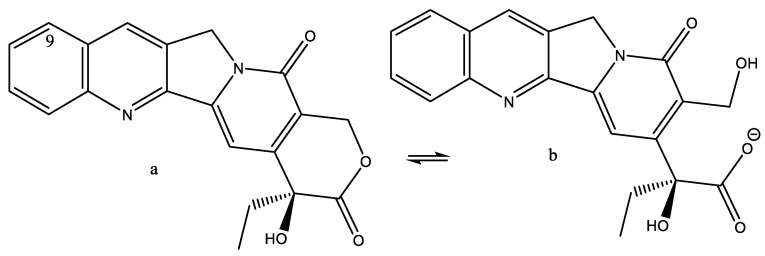
Closed and open forms of camptothecin.

Diffusion experiments, such as diffusion ordered spectroscopy (DOSY) [31], may be used to estimate the size of molecules, or, more importantly, of molecular complexes. Camptothecin is a natural product isolated from the stem wood of *Camptotheca acuminate*. It is a topoisomerase I inhibitor and is, as such, used in cancer therapy. Camptothecin may exist both in the lactone form/Figure 7a and in an open form/Figure 7b) in equilibrium at physiological pH [32].

**Figure 8:**
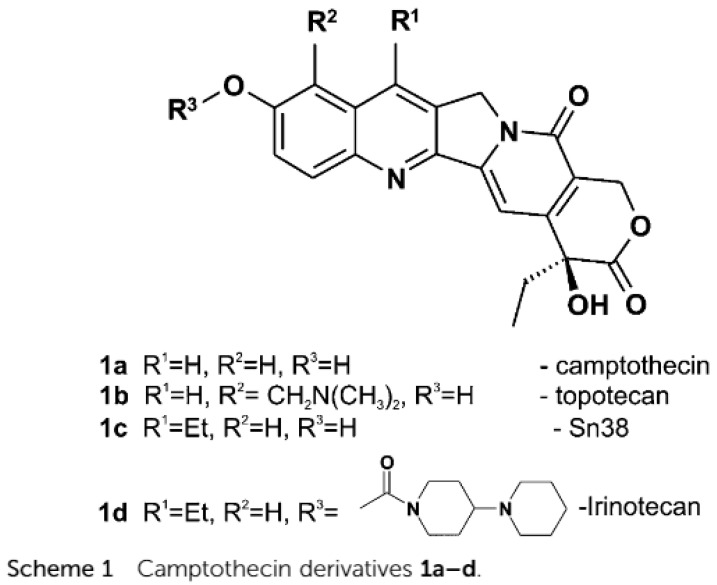
Camptothecin and derivatives. Taken from Ref. [32], with permission from *WILEY-VCH Verlag*.

**Figure 9:**
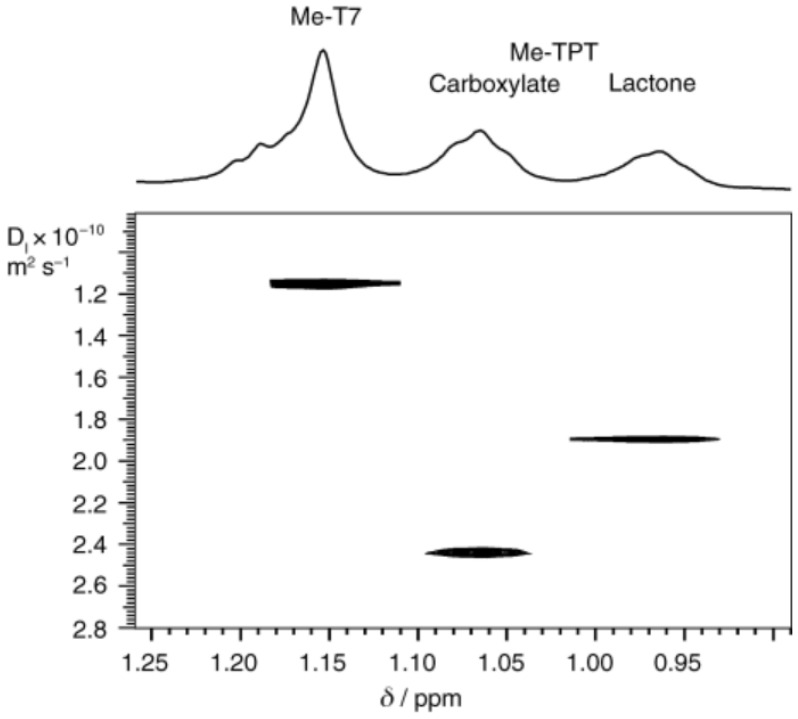
A DOSY spectrum showing diffusion coefficient versus methyl chemical shift for Topotecan with a nicked decamer. Taken from Ref. [32], with permission from *WILEY-VCH Verlag*.

A very close relative is topotecan (R^2^ = CH_2_N(CH_3_)_2_ in Figure 8. The difference in binding between the open and closed forms is demonstrated in the DOSY experiment in Figure 9 The diffusion coefficient is related to the hydrodynamic radius of the molecules.

It is seen that the carboxylate form forms a weaker complex with the decamer-DNA than the lactone form.

Derivatives such as Sn38 and irinotecan have shown great potential in cancer treatment. Other derivatives are shown to alkylate DNA [33,34].

## 6. Computer Aided Structure Analysis

**Figure 10:**
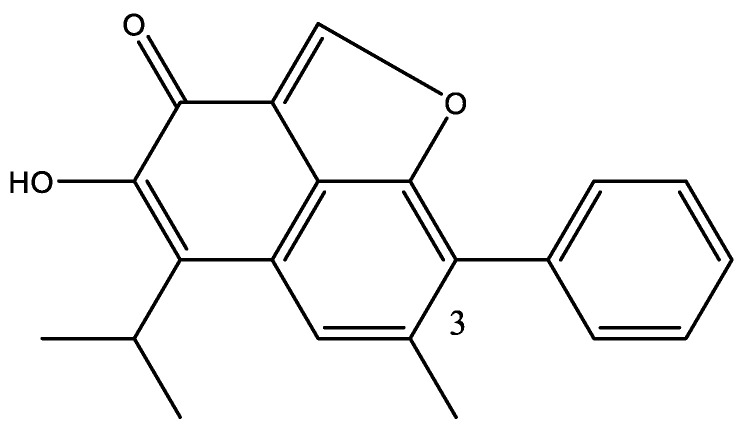
Dehydrogossypol.

**Figure 11:**
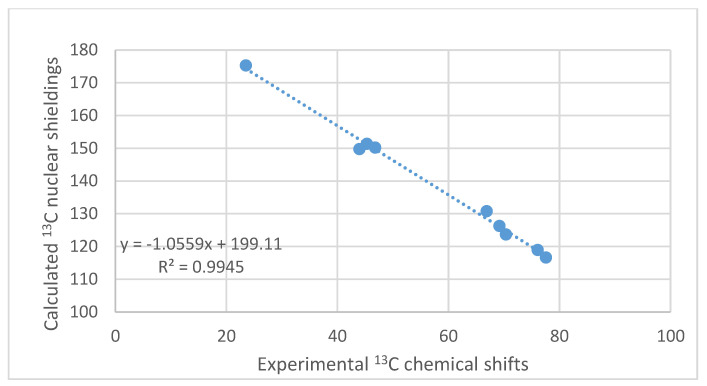
Plot of calculated ^13^C nuclear shieldings vs. experimental chemical shifts of anhydrogossypol (see Figure 10). ^13^C NMR data from Ref. [45].

Calculations are a useful tool in structure elucidation of natural products [35,36]. Comparison with calculated mostly ^13^C chemical shifts can also be used to correct structures [37]. Several methods have been developed, DU8+ [38], DP4 [39] and DP4+ [40]. These techniques are nicely reviewed [41]. Calculations of nuclear shieldings using even simple basis sets can be used to evaluate tautomerism. An example of a calculation of the unusual dehydrated gossypol is shown in Figure 10. The nuclear shieldings are calculated using the B3LYP functional [42] and the simple G(d) Pople basis set [43], using the Gaussian program package [44]. Gossypol is a dimer, but with the methyl group in position 3, the two units are almost perpendicular, so the second unit can be mimicked by a phenyl ring. All carbon except C-2, C-3 and C-4 are used in the plot. A reasonable correlation is found (see Figure 11).

## 7. Examples

Obviously, many natural products are glucosides. However, in this review the focus will be on the aglycone, as the NMR of carbohydrates is treated in another contribution. 

### 7.1. β-Diketones

For a β-diketone system, see Figure 3. β-diketones exist typically both on the enol (a,b) and keto form (c).

#### Curcumins

**Figure 12:**
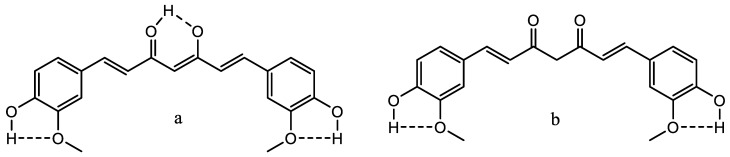
The two tautomers of curcumin. (**a**) is the enol-form, (**b**) the keto-form.

Curcumin and derivatives thereof have attracted much attention recently [46,47,48]. They are a classic, extended β-diketone system. Curcumin is usually isolated together with the demethoxy and the bis-demethoxy derivatives. They exist as a mixture of the keto (b) and the enol form (a) of Figure 12.

The amounts of the two tautomeric forms, the enol and the keto form, can easily be determined by ^13^C NMR [49]. The enol form shows fast interconversion of the OH-proton. The presence of the phenolic OH protons causes exchange of the OH protons, leading to difficulties in detecting all OH protons by ^1^H NMR. The biological effects of the two tautomeric forms are discussed in relation to Alzheimer’s disease [50]. 

### 7.2. β-Triketones Are Typically also Tautomeric Systems

An example is tetramic, tetronic and thioteramic acids. This is a very large group of natural products. Their biological effects have been reviewed [51]. 3-acyltetramic acid derivatives have been synthesized and analysed by NMR [52,53]. In such a system, two tautomeric equilibria may be present. This type of system has been reviewed [54].

#### 7.2.1. Tenuazonic Acid

**Figure 13:**
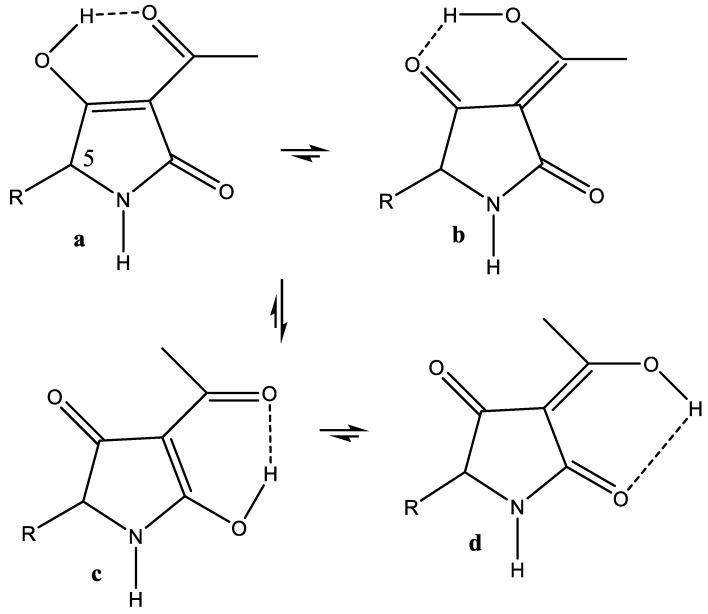
Tautomers of tenuazonic acid, R = isobutyl.

The same simple approach can be used for a tautomeric system, as shown below. Tenuazonic acid is a eukaryotic protein synthesis inhibitor. NMR data for the isopropyl derivative, R = isopropyl, are given in [55]. Two different sets of signals are observed in the ^13^C NMR spectrum, because the equilibrium between a–b and c–d is slow (Figure 13). The authors describe the compounds as the b and c forms, with the b form being the dominant one. Calculating the nuclear shieldings for form a and form b and correlating those with the observed chemical shifts in the same way as demonstrated in Figure 11, it turns out that the best correlation, R^2^ = 0.9981, was obtained at ~30% of a and 70% of b (see Figure 13). In case of the c–d equilibrium, the same type of calculation gave 10% of c and 90% of d. If an equilibrium is at play, this could be determined by measuring deuterium isotope effects at ^13^C chemical shifts, as it was conducted for the similar 3-acyltetronic acids [56].

Data for the benzyl derivative, R = CH_2_Ph, are very similar to the ones with R = isopropyl, except, of course, for C-5. For tenuazonic acid, R = sec. butyl, the calculated nuclear shieldings are very similar to those of the derivative with R = isopropyl. One can then assume that this will behave similarly.

#### 7.2.2. Usnic Acid

Usnic acid can exist both as + and − forms, due to the chiral carbon, 9b. Usnic acids are isolated from lichens and have antimicrobial, antiviral, anticancer and anti-inflammatory properties [57]. Usnic acid has three different regions—the strong intramolecular hydrogen bond of ring A, a weak hydrogen bond between the OH group at position 9 and the carbonyl group at position 1. The third and important feature is the formal triketone system of ring C.

**Figure 14:**
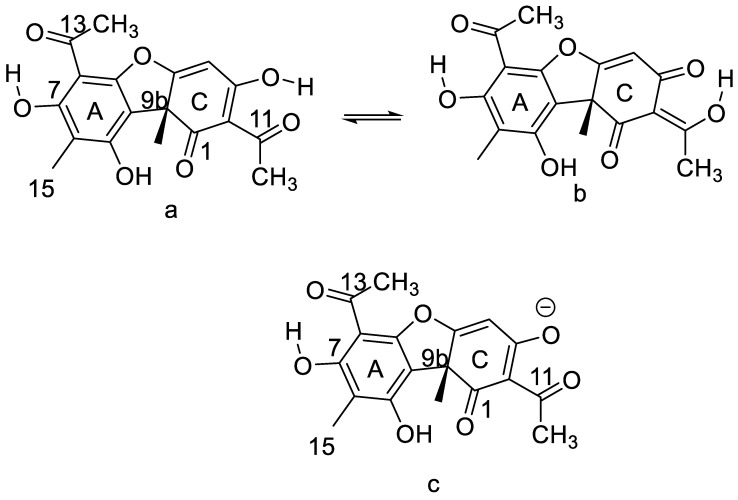
Tautomeric structures of usnic acid (**a**,**b**) (**top**) and the anion (**c**) (**bottom**).

Sometimes the C-ring is given at the keto-form, but this is clearly wrong, as the two-bond deuterium isotope effects at C-3 and C-11 both are ~0.5 ppm [14]. This shows that a tautomeric equilibrium between the enol-forms (Figure 14a,b) with an equilibrium constant close to 1 is present in CDCl_3_. One of the unfortunate features of usnic acid is the low solubility in water [58]. This was partially improved by adding a polyether at the OH-7 group [23]. Another feature is a low pKa value, due to the triketone system. This was in a mixed water system determined as 4.4. [59]. The pKa value in a more water-like environment was determined as 4.3, using the pegylated usnic acid. This means that at physiological pH, usnic acid will be an anion (Figure 14c) [23].

**Figure 15:**
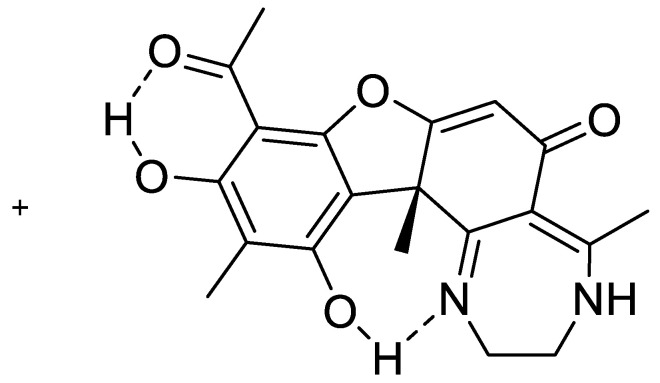
Usnic acid with cyclic extension. From Ref. [63].

Derivatives of usnic acid are typically Schiff bases reacted at the carbonyl carbon, C-11. In some cases, both C-11 and C-12 are involved, leading to cyclic compounds [60,61,62,63]. An example of the latter is a derivative (see Figure 15), which shows promising results in an in vivo test against a rare brain cancer. As the disease is rare, this derivative, even if it shows promising test results, will never be developed into a drug.

#### 7.2.3. Tetracyclines

Tetracyclines are long known antibiotics. Tetracycline, chlortetracycline, oxytetracycline and demeclocycline are all natural products. A number of modified tetracyclines do also exist [64].

**Figure 16:**
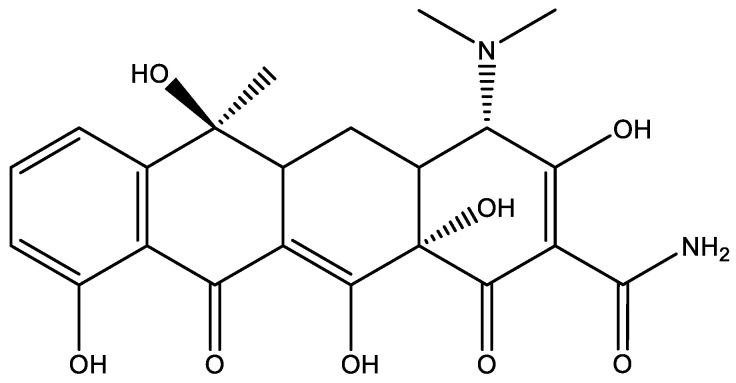
Tetracycline.

**Figure 17:**
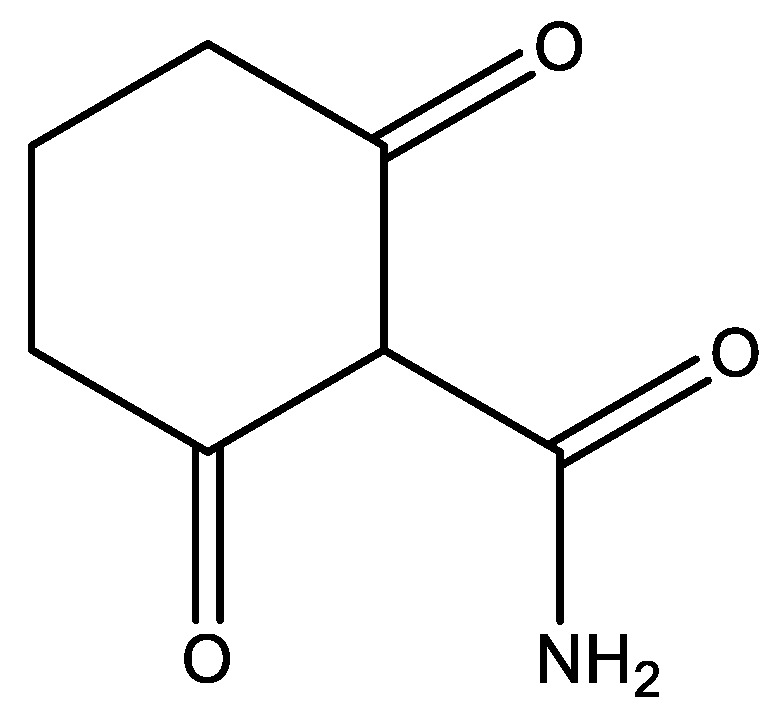
2-carbamoyl-1,3-cyclohexanedione shown on the keto-form.

Tetracycline has a complex structure, which may not be the same in solution as the structure in Figure 16. At the bottom of the molecule is a β-diketone system with the possibility of hydrogen bonding, including the phenol OH (for a model compound, see Figure 3). To the right is a system corresponding to 2-carbamoyl-1,3-cyclohexanedione (Figure 17).

In addition, tetracycline does also contain an amino group, so the structure can be different, probably protonated, depending on the pH. In order to elucidate the structure, different techniques have been used, ^18^O isotope effects on ^13^C chemical shifts, deuterium isotope effects on ^13^C chemical shifts and comparison with model compounds, such as the ones in Figure 17, and of *o*-hydroxydibenzoylmethane (see Figure 3) [14].

#### 7.2.4. Garcinia Derivatives

**Figure 18:**
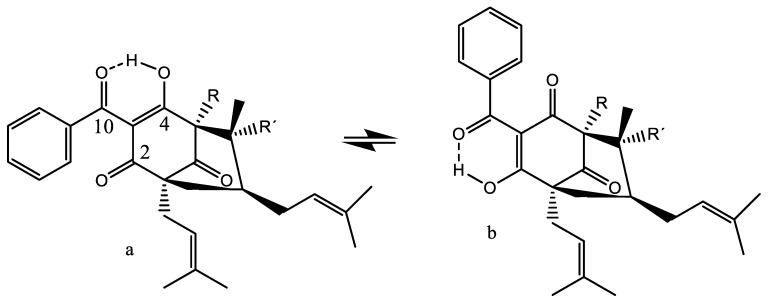
Tautomerism between the (**a**,**b**) forms. Structures of R,R′ = H, garciniaphenone and R = prenyl, R′ = H, 7-*epi*-clusianone.

For garciniaphenone, two different tautomers were observed, as seen in Figure 18 a,b. The OH resonances were observed as 17.90 and 17.35 ppm, showing strong hydrogen bonds, but also showing that the equilibrium between the two tautomers is slow on the NMR time scale. The two tautomers exist in a ratio of 5:1 for a/b. A similar behaviour was found for guttiferones A and E, clusianone and 7-*epi*-clusianone [65,66,67]. 

Guttiferone A, (1R,5R,7R,8S)-(+)-3-(10-(3,4-dihydroxyphenyl)-10-hydroxymethylene)-8-methyl-1,5,7-tris(3-methyl-2-butenyl)-8-(4-methyl-3-pentenyl)-bicyclo [3.3.1]nonane-2,4,9-trione (see Figure 18a, R = R’ = prenyl; the phenyl ring is 3,4-dihydroxy) shows HIV-inhibitory properties [68] and, being a β-triketone, it is shown to exhibit tautomerism involving the OH group at C-10 and the carbonyl group at C-4. However, the OH resonances of the ^1^H NMR spectrum integrate 3H at 11.02 ppm in pyridine-d_6_. This is due to a fast exchange between the OH protons, but is in agreement with an average chemical shift caused by 15–17, 9 and 9 ppm, typical values for β-di- or triketones and non-hydrogen bonded phenols.

The observation that tautomerism leads to a conformational change is interesting and of great importance for the biological action.

Guttiferone A, Garciniaphenone and 7-epiclusianone are discussed [69], together with many other natural products, as cathepsin inhibitors [70]. However, as it is often seen, only one tautomeric form is given, despite the big difference between the two tautomers (Figure 18).

### 7.3. Flavonoids

**Figure 19:**
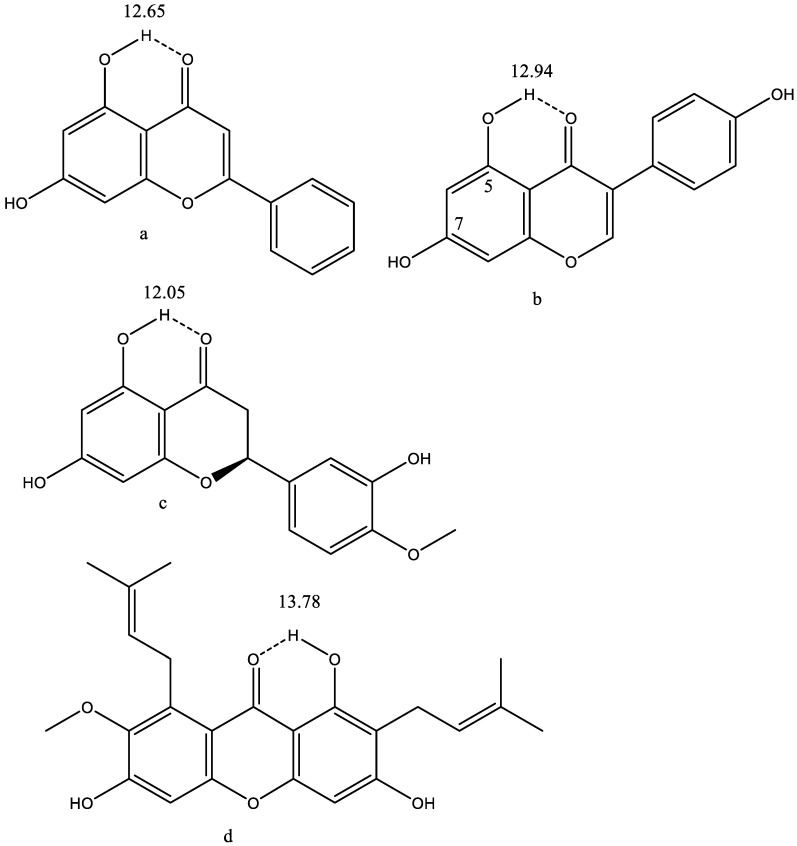
Flavonoids. Numbers are OH chemical shifts. (**a**). Chrysin. Data from Ref. [73]. (**b**). Genistein. From Ref. [74]. (**c**). Hesperetin. Data from the SDBS data base [75]. A number of other compounds are given in Ref. [76]. (**d**). α-mangostin from Ref. [77].

Many flavonoids are potential drugs. A list can be found at the Drugbank [71]. A large group has tested for anticancer properties in a docking study [72]. Typical examples are seen in Figure 19.

Genistein (Figure 19b) is considered for the treatment of prostate cancer. It has been suggested that a complex with cyclic amines, such as piperazine or morpholine, should give better biological effects [78]. It was found, by studying deuterium isotope effects on ^13^C chemical shifts, that the complex is formed with the OH-group in position 7, because the one in position 5 forms a strong hydrogen bond, so this is not available. The OH proton of the OH group in position 7 is partially transferred to the nitrogen of the complexing agent [79].

Genistein was found in a data base docking study to bind to the estrogen receptor-b(ERb) [80,81].

The mangostins (α, β and γ) are isolated from the mangosteen fruit. The α-mangostin show antioxidant, antimicrobial, anti-inflammatory, antifungal and antibacterial effects [82].

### 7.4. Rifampicin

**Figure 20:**
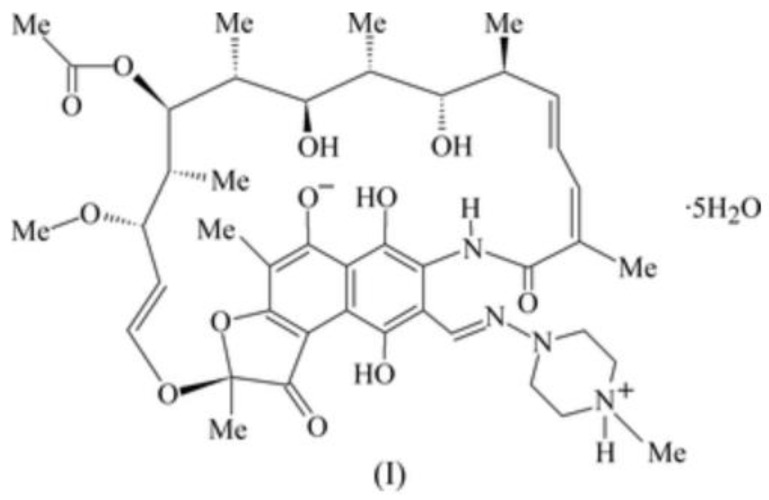
Structure of rifampicin taken from Ref. [85]. Reproduced with permission of the International Union of Crystallography.

Rifampicin is an antibiotic used against tuberculosis. From NMR studies, it was shown to be a zwitter ionic form, as seen in Figure 20. The OH-8 proton is transferred to the piperazine nitrogen in protic solvents [83] and in DMSO. The resulting O^−^ leads to a very strong hydrogen bond with an OH chemical shift of 15.8 ppm in DMSO-d_6_. This is similar to the study of the anion of naphthalene-4,5-dihydroxy-2,7-disulphonate [84]. However, it would be good to know if the OH proton is permanently at the C-1 carbon. Deuterium isotope effects at ^13^C chemical shifts could help elucidate this question. The zwitter ionic structure is important for the high antibiotic activity [83].

### 7.5. Gossypol

**Figure 21:**
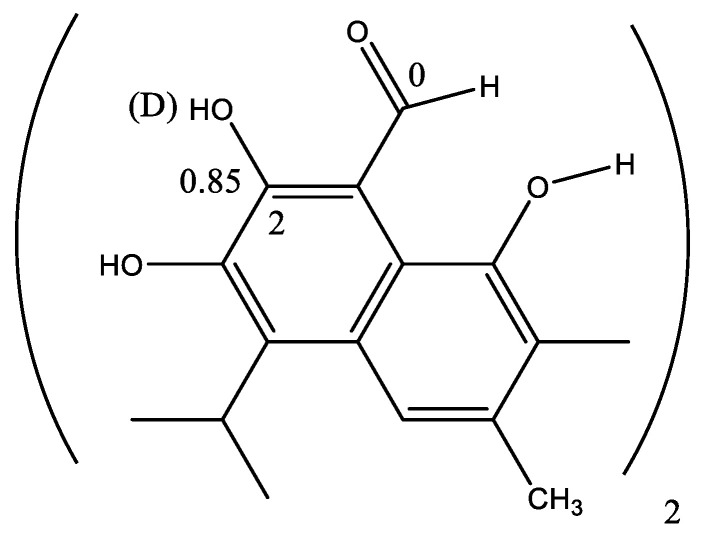
Gossypol. Selected deuterium isotope effect on ^13^C chemical shifts in ppm. Data from Ref. [87].

Gossypol (Figure 21) is isolated from cotton and has been tested as a male contraceptive. The structure has been suggested to be tautomeric [86]. A large deuterium isotope effect at C-2 was observed early on [87]. Based on the fact that no effect was seen at the aldehyde carbon, a tautomeric equilibrium seemed less likely. A resonance structure involving the OH group in position 8 was suggested [88], but steric compression could also be a factor leading to the large isotope effect at C-2 [89].

### 7.6. Hymenialdisine (Hys)

**Figure 22:**
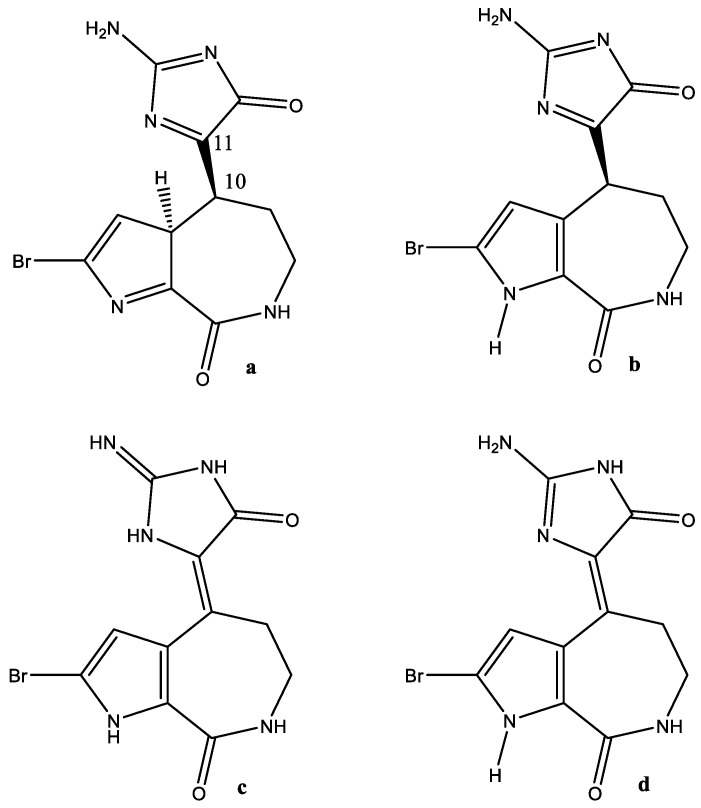
Different presentations of hymenialdisine. The odd form (**a**) is from Ref. [95].

Hymenialdisine is a guanidine-containing natural product that is known as a kinase inhibitor. It can exist both as an E- and a Z-form (in Figure 22c,d the Z-form is shown, the E-form is obtained by rotation around the 10–11 bond). A debromo form, as well as analogue structures, are also found [90]. In an early study, Williams et al. [91] reported that Hys could be tautomeric, as seen in Figure 22c,d. The structure varies in different papers (NH_2_ vs. NH) [92]. Docking studies were performed assuming the NH structure (Figure 22c) [93]. Hys was also docked to cyclin-dependent kinases assuming the same structure [94]. 

### 7.7. Pratensilins

**Figure 23:**
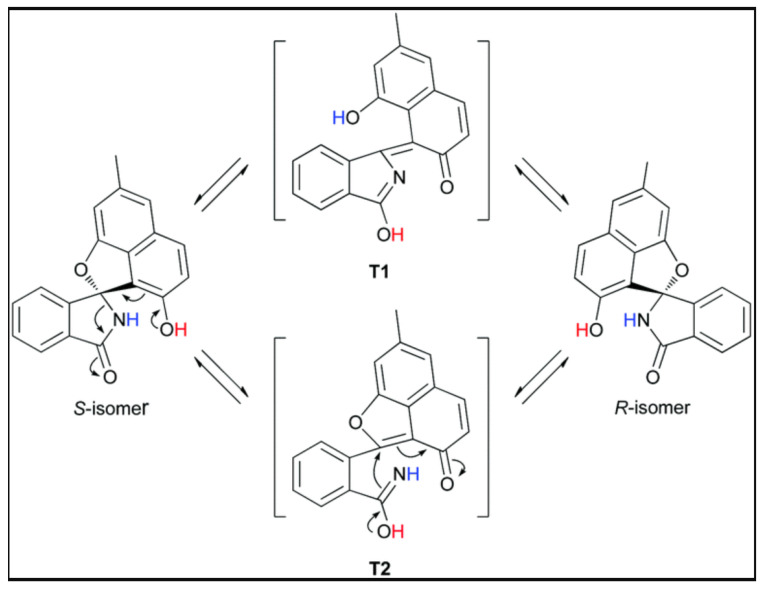
Conversion of a + to − form of pratensilins A due to tautomerization. Taken from Ref. [96], with permission from the Royal Society of Chemistry.

An interesting possibility is the inversion of a stereo center caused by tautomerism, as seen in the Pratensilins isolated from the marine *Streptomyces* sp. The formula seen in Figure 23 showed moderate cytotoxicity towards eight human cancer cell lines [96].

### 7.8. Thiotropocin

**Figure 24:**
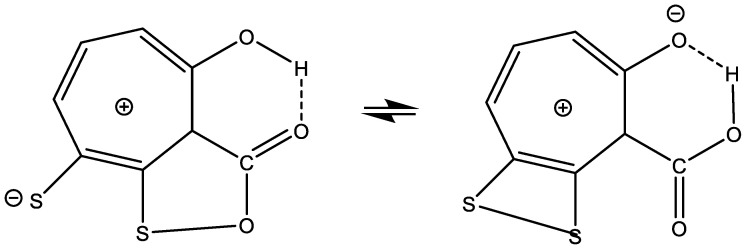
Tautomerism of Thiotropocin. From Ref. [97].

Thiotropocin is isolated from the *Roseobacter* strain 27-4 and shows antibiotic properties. It was originally assumed that both forms of Figure 24 existed. However, recently, it has been shown that they are “tautomers” or, as coined by the authors, structural isomers [97]. For a discussion of the biological effects, see Ref. [98].

### 7.9. Prodigiosins

**Figure 25:**
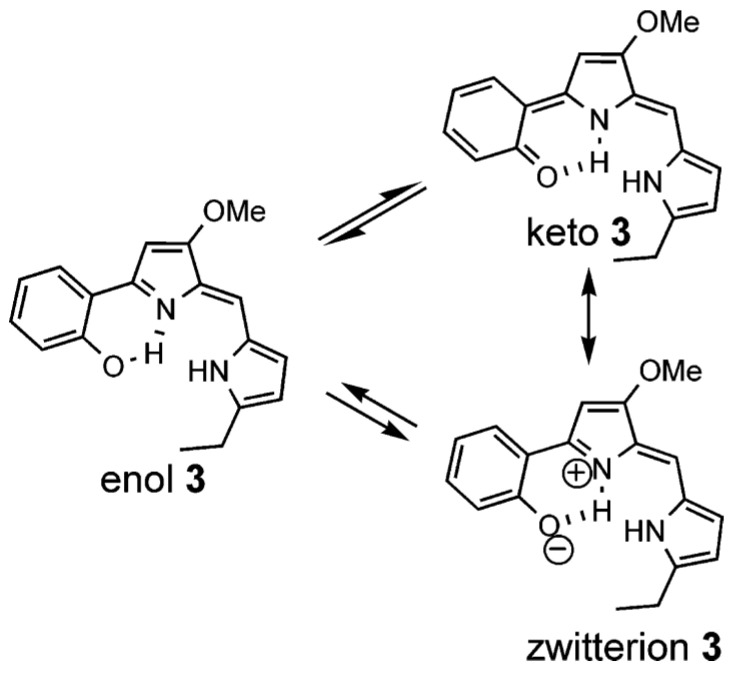
Tautomerism of a Prodigiosin. Taken from Ref. [99], with permission from the American Chemical Society.

Prodigiosin is isolated from *Serratia*, *Streptomyces* and *Bacillus* strains. Prodigiosin itself is toxic, but derivatives are found to have a large number of important biological properties, such as immunosuppressive, antimicrobial, antimalarial and anticancer properties. As seen in Figure 25, tautomerism is present when a phenolic proton is present. OH chemical shifts are as high as 14.6 and 11.5 ppm for the OH and NH protons, respectively [99].

### 7.10. Muscimol

**Figure 26:**
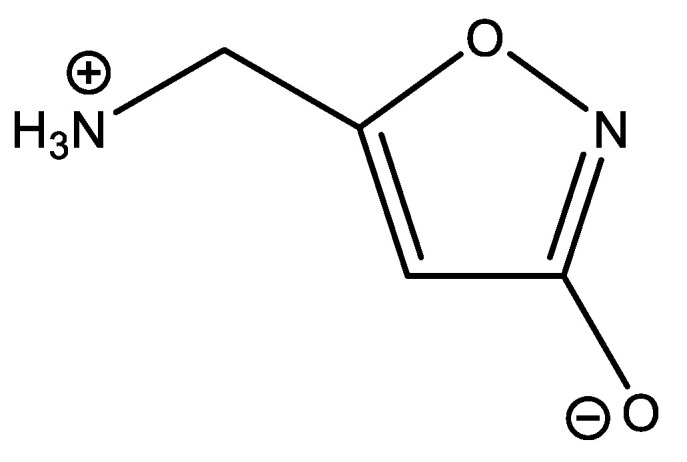
Muscimol.

Calculations have also been used to show that muscimol exists as a zwitter ion in water (Figure 26). In this case, a high basis set 6-311++G-(3df,2pd) has been used [100]. This is necessary, because of the negative charge. Muscimol was considered as a model for the neurotransmitter γ-aminobutyric acid (GABA).

### 7.11. Hypericin

**Figure 27:**
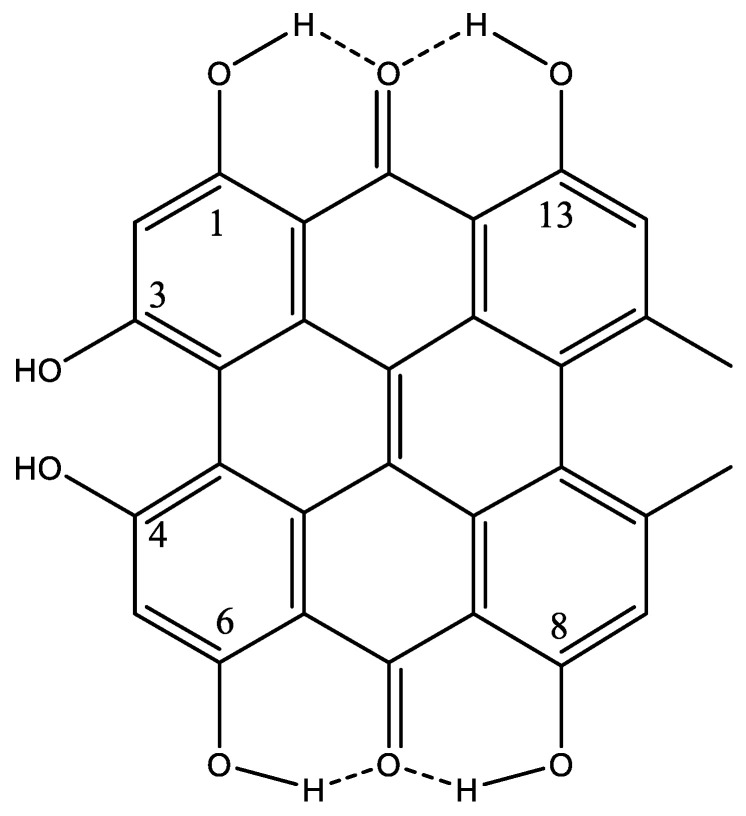
Hypericin.

Hypericin is isolated from St. John’s wort and is a powerful natural photosensitizer used in photodynamic therapy [101]. Hypericin is a typical system with two hydrogen bond donors to the same acceptor (see Figure 27). The proton chemical shift of OH-1,6 is 14.8 ppm and that of OH-8,13, is 14.2 ppm. For pseudohypericin, similar values were obtained [102]. For the bay protons (OH-3,4) in acetone, the OH chemical shifts are reported as 11.6 ppm. DFT calculations have been used to confirm the structure in acetone [103]. As the bay protons are very acidic, pK_a_ 1.7–2.0, an anion has been suggested in DMSO-d_6_, resulting in a chemical shift of 17.2–17.5 ppm. This points to a very strong hydrogen bond. This has also been confirmed by DFT calculations [103]. The high acidity was explained by the vinylogous carboxylic acid nature of the hydroxyl groups [104]. However, this low pK_a_ value suggests an anionic structure at physiological pH similar to that obtained in DMSO.

### 7.12. Xanthohumol

Xanthohumol is found in hops and, therefore, in beer. However, only in small amounts, as it is converted into other compounds during the brewing process. It has multiple bioactivities, including anticancer, antidiabetic, antibacterial, anti-inflammatory activities [105]. The hydrogen bond is clearly defining the structure of the molecule. The strength of the hydrogen bond can be estimated from its OH chemical shift [106]. With a predicted value of 12.04 ppm, the hydrogen bond energy can be predicted as 7.35 Kcal/mole.

**Figure 28:**
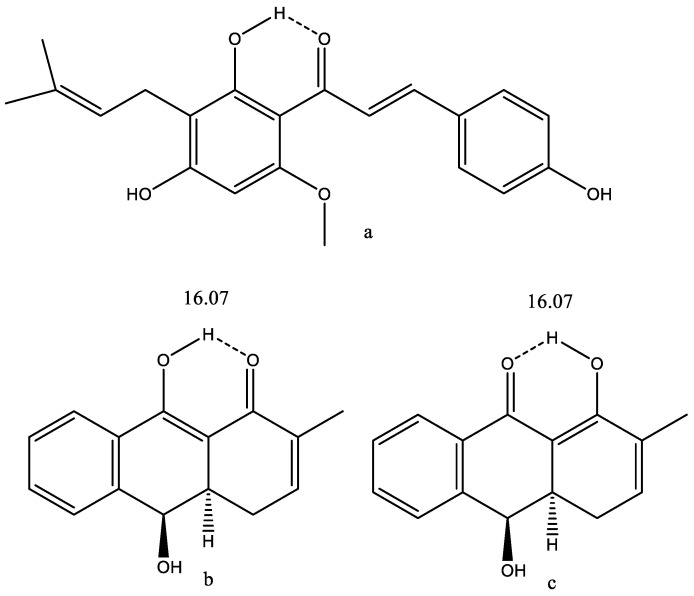
(**a**) A hydrogen bonded version of xanthohumol. (**b**,**c**) tautomeric forms ofDihydroanthracen-1(4*H*)one.

**Figure 29:**
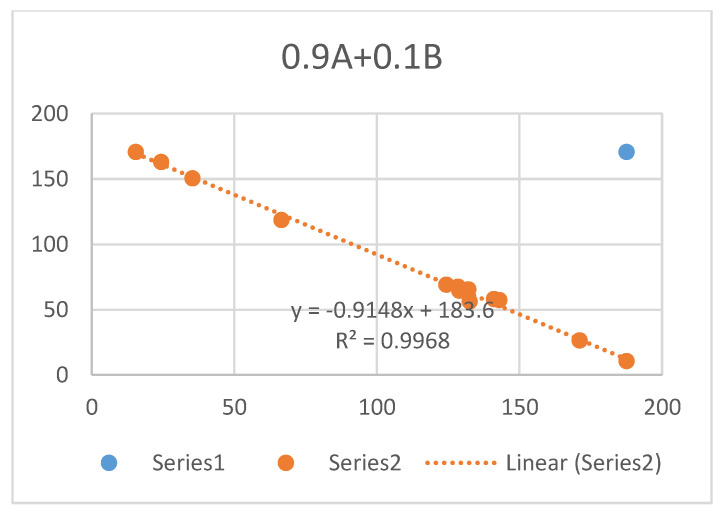
Plot of experimental ^13^C chemical shifts vs. ^13^C calculated nuclear shieldings, B3LYP, 6-31G(d).

Compound b of Figure 28 displayed moderate soluble epoxide hydrolase (sEH)-inhibitory activity. It is isolated from *Rubia philippinensis.* The OH chemical shift is very high and a very small OH temperature coefficient was found. This structure has all the features necessary for being tautomeric. The authors speculated, based on HMBC data, that the compound could be tautomeric. They measured a ^13^C NMR spectrum at 200 K and, as they did not find two sets of signals, they concluded that no tautomeric equilibrium took place [107]. However, the barrier to interconversion in β-diketones is normally so low that two tautomers cannot be found. A calculation of ^13^C nuclear shieldings and a comparison with experimental ^13^C chemical shifts (see Section 6 for an example) shows a slight improvement when assuming 90% of b and 10% of c. The increase from pure b to a tautomeric mixture is from R^2^ = 0.9965 to R^2^ = 0.9968 (see Figure 29). Furthermore, the energies of the two structures do only differ by 2.6 KJ based on DFT calculations, B3LYP 6-311++G(d,p), and with b preferred. 

### 7.13. Sunflower Trypsin Inhibitor

**Figure 30:**
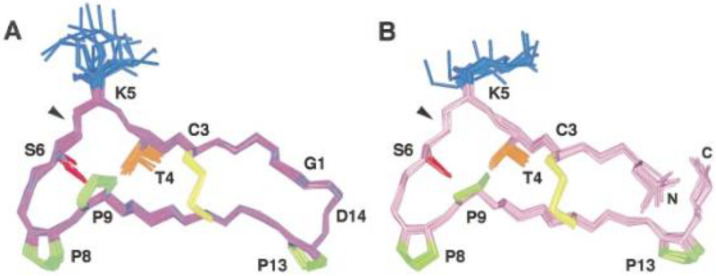
NMR structures of sunflower trypsin inhibitor, closed (**A**) and open (**B**) forms. Yellow color marks a disulfide bridge. Letters are amino acid abbreviations. From Ref. [108], with permission from Elsevier.

As the name says, this is a trypsin inhibitor isolated from sunflowers. It is a cyclic peptide (Figure 30) existing in a closed and an open form. The following NH protons are found to be involved in hydrogen bonds, G1, R2, T4, I10 and F12 and R2, T4, I10 and F12, in unbound cyclic and acyclic SFTI-1, respectively. The many hydrogen bonds are important for the tight structure of the peptide and for its biological function. The hydrogen bonds lead to similar structures for the closed and the open forms [108].

### 7.14. Humic Substances

**Figure 31:**
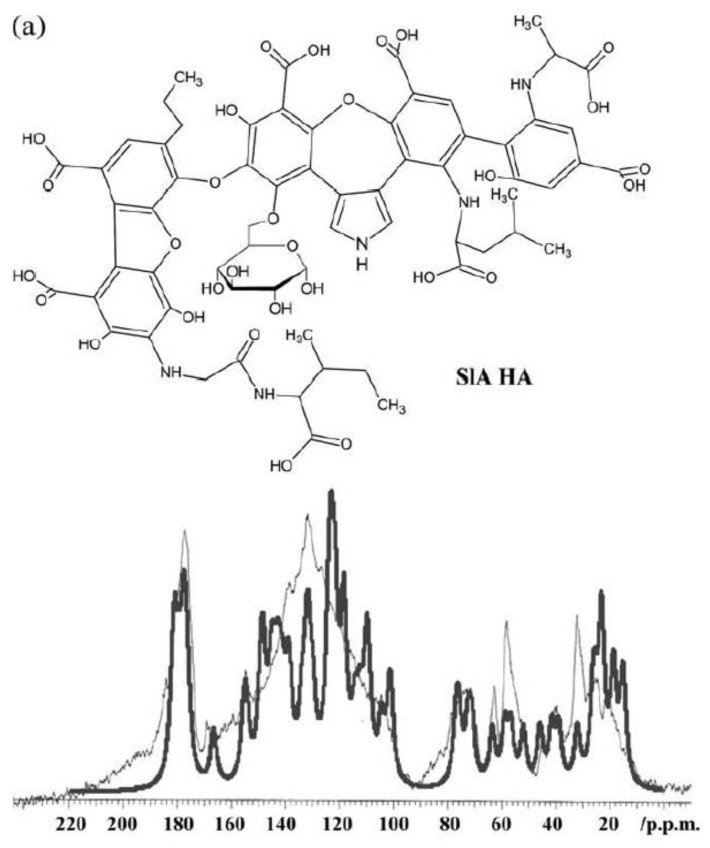
Top (a). Structural elements suggested for humic acid. Bottom. Spectrum behind (thin line) is the ^13^C NMR spectrum in solution. Spectrum in front is the predicted ^13^C NMR spectrum based on the shown structure and database data. Taken from Ref. [115], with permission from John Wiley and Sons.

Humic and fulvic acids are degradation products of plant material and may be isolated from soil. Humic acid is used to stimulate the immune system, used against viral infections and used for drug delivery [109,110]. The structure is very complex, but somewhat constant when isolated from podzols. Simpson et al. compared 2D spectra of similar carbohydrates and proteins [111,112,113]. Another method to predict the structural elements is to reconstruct ^13^C NMR spectra using data from databases. A great number of different structures have been proposed. The reconstruction of the ^13^C NMR spectra was then used to estimate the likeliness of some of these structures [114]. The structural element shown in Figure 31 shows possibilities of hydrogen bonding, something which has not yet been explored.

## 8. Conclusions

This review has discussed how rather simple NMR measurements can help in determining hydrogen bonds, the presence of tautomerism or the occurrence of ionic or zwitter ionic natural compounds. Hydrogen bonding may be important in defining the structure of molecules, in the sense that side-chains are fixed and the hydrogen bond energies can be quite high. Depending on the type, the degree of how different the two tautomers are varies quite considerably, ranging from enol–enol tautomerism, keto–enol tautomerism, tautomerism involving NH protons, conformational changes to what is termed “structure isomers”. For most tautomeric compounds, the barrier between the two forms is often not known, which makes it difficult to assess whether both forms (usually two but in some cases more) should be taken into account in the evaluation of biological effects. A classic example is curcumin, for which both the enol form and the keto form can be observed and have different biological functions. Often tautomerism is not detected, or it is dismissed, based on incomplete information and only one structure is given. In those cases, the measurement of isotope effects on chemical shifts is an invaluable tool to obtain correct information. Tautomeric compounds are difficult to represent in chemoinformatics systems. This clearly poses a problem in using information systems for biological effects of tautomeric compounds and in docking experiments. Doing docking only with the major tautomer could lead to wrong conclusions. However, for the non-tautomeric compounds docking studies have shown that many natural products may fit into the active sites of enzymes, DNA, etc.

## Data Availability

Not applicable.
